# GLIM criteria using NRS-2002 and MUST as the first step adequately diagnose the malnutrition in Crohn’s disease inpatients: A retrospective study

**DOI:** 10.3389/fnut.2022.1059191

**Published:** 2023-01-11

**Authors:** Meng Wang, Qin Guo, Hong Liu, Min Liu, Chenyi Tang, Jinru Wu, Guo Feng, Wei Wu

**Affiliations:** ^1^Department of Clinical Nutrition, The Third Xiangya Hospital, Central South University, Changsha, Hunan, China; ^2^Department of Gastroenterology, The Sixth Affiliated Hospital of Sun Yat-sen University, Guangzhou, Guangdong, China; ^3^Department of Gastroenterology and Urology Medicine, Hunan Cancer Hospital, The Affiliated Cancer Hospital of Xiangya School of Medicine, Central South University, Changsha, Hunan, China

**Keywords:** Crohn‘s disease, nutritional risk, malnutrition, nutritional risk screening 2002, Malnutrition Universal Screening Tool (MUST), Global Leader Initiative on Malnutrition (GLIM), ASMI, FFMI

## Abstract

**Objective:**

The Global Leader Initiative on Malnutrition (GLIM) criteria have been recommended for malnutrition diagnosis recently, for which the first step is malnutrition risk screening with any validated tool. This study aims to investigate the incidence of nutritional risk and malnutrition in Crohn’s disease inpatients and compare the suitability of Nutritional Risk Screening 2002 (NRS-2002) and Malnutrition Universal Screening Tool (MUST) as the first-step screening tool for GLIM criteria.

**Methods:**

We retrospectively analyzed the clinical data of Crohn’s disease inpatients in our hospital from August 2016 to December 2019. NRS-2002 and MUST were used for nutritional screening at the time of admission. GLIM and Patient Generated-Subjective Global Assessment (PG-SGA) were used for malnutrition assessment, respectively. Patients without nutritional risk screened by NRS-2002 but with malnutrition risk screened by MUST were especially screened out. The appendicular skeletal muscle mass index (ASMI), fat-free mass index (FFMI), body fat percent (BFP), and body cell mass (BCM) were measured by the Biospace Inbody S10 composition analyzer.

**Results:**

A total of 146 Crohn’s disease patients were enrolled, of which 62.3 and 89.7% had nutritional or malnutrition risk according to NRS-2002 and MUST, respectively. The prevalence of malnutrition assessed by GLIM was 59.6% (87 cases) and 82.2% (120 cases) when NRS-2002 and MUST were used as the first step of GLIM respectively. Meanwhile, 99 patients (67.8%) had malnutrition when assessed by PG-SGA. There were 41 patients who were not at nutritional risk according to NRS-2002 but were at malnutrition risk determined by MUST. At last, 33 patients were GLIM-defined, and 16 patients were PG-SGA-defined malnutrition among the 41 patients.

**Conclusion:**

The nutritional risk or malnutrition is common in Crohn’s disease inpatients. It is recommended to use a variety of nutritional assessment tools for Crohn’s disease inpatients. MUST can be used as a good supplement for the patients with a score of NRS-2002 lower than 3 in order to decrease the miss rate of GLIM-defined malnutrition.

## 1. Introduction

Crohn’s disease (CD) is a chronic, systemic autoimmune, transmural inflammation disease of the whole intestine. Reduced nutrient absorption, increased nutrient requirements, gastrointestinal nutrient losses, and chronic inflammation exposure increased the risk of malnutrition in CD patients (1–3). Therefore, the nutritional status assessment of these patients becomes much more important. Currently, the main nutritional assessment tools or parameters are used including body mass index (BMI), Nutritional Risk Screening-2002 (NRS-2002), and Patient-Generated Subjective Global Assessment (PG-SGA) (4–8). The Asian diet and inflammatory bowel disease (IBD) working group emphasized that weight or BMI alone cannot accurately predict the nutritional status of the patient (4); unfortunately, they did not also clearly indicate the appropriate nutrition assessment method for IBD patients.

According to the Global Leadership Initiative on Malnutrition (GLIM) 2019, the diagnosis of malnutrition can be divided into two steps: screening and assessment. First, screening to identify nutritional risk status by using any validated screening tool, and second, assessment for diagnosis and classification of the severity of malnutrition (at least 1 phenotypic criterion and 1 etiologic criterion should be included) (9). The ESPEN guidelines advised to use any validated tool as the first step of GLIM but without any further recommendation (9). As a recognized nutritional risk screening tool for inpatients, NRS-2002 is default as the first step of GLIM. In principle, the second step of GLIM can not be continued once NRS-2002 shows no nutritional risk. Our previous study found that patients with a negative NRS-2002 result may also had a low muscle mass (6), suggesting that some patients may have nutritional risk or even malnutrition that can not be screened out by NRS-2002 (10). Should other validated tools be used for the first step of GLIM criteria as nutritional risk screening? The Short-form Mini-nutritional Assessment (MNA-SF) or Malnutrition Universal Screening Tool (MUST) (11) is also a validated malnutrition risk screening tool. The former is commonly used to assess the malnutrition risk of older adults (12–15), while the latter is thought to be consistent and reliable for different evaluators in different settings and has good reproducibility (16).

Therefore, we used NRS-2002 and MUST for nutritional screening, respectively, and used GLIM and PG-SGA for malnutrition assessment, respectively, to investigate the significance of various nutritional risk screening and malnutrition assessment tools in the nutritional diagnosis of CD patients.

## 2. Materials and methods

### 2.1. Study design

This study retrospectively analyzed the clinical data of Crohn’s disease inpatients in our hospital from August 2016 to December 2019. Participants were patients with Crohn’s disease admitted to the Department of Gastroenterology in the Third Xiangya Hospital, Central South University, during that period. This study has been approved by the Institutional Review Boards of the Third Xiangya Hospital, Central South University (2019-S540). A waiver of informed consent has been obtained for this retrospective study.

### 2.2. Subjects

The inclusion criteria were as follows: (1) The patients were 18-90 years old. (2) CD was diagnosed according to the Montreal classification (17). According to the age of disease onset, lesion site, and disease behavior, the patients were divided into groups of A1 (not older than 16 years), A2 (17–40 years old), and A3 (> 40 years old); L1 (ileal), L2 (colonic), L3 (ileocolonic), and L4 (isolated upper disease); B1 (non-penetrating, non-stricturing), B2 (stricturing), B3 (penetrating), and P (perianal disease modifier). (3) The patients were conscious and had no serious edema and ascites. (4) The height and the body weight could be accurately measured. (5) Length of hospital stay was longer than 24 h, and surgery was not performed during the hospitalization.

### 2.3. Methods

**FIGURE 1:**
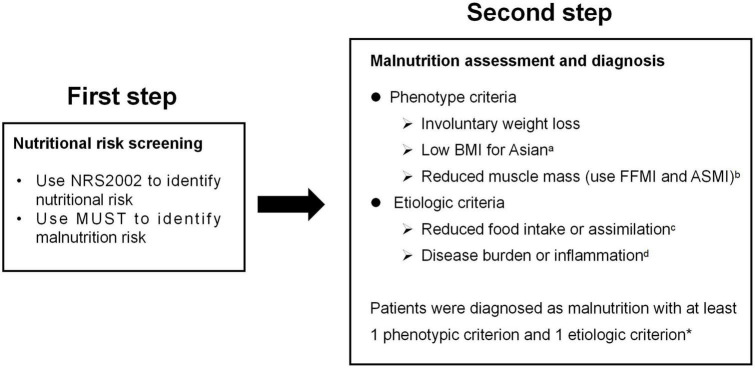
GLIM diagnostic scheme for screening, assessment, and diagnosis of malnutrition in our study. *^a^*BMI < 18.5 kg/m^2^ if < 70 years or < 20 kg/m^2^ if > 70 years; *^b^*FFMI < 15 kg/m^2^ for women, < 17kg/m^2^ for men, or ASMI < 5.7 kg/m2 for women, < 7.0kg/m^2^ for men; ^c^ < 50% of energy requirements > 1 week, or any reduction for at least 2 weeks, or any chronic gastrointestinal symptoms that lead to inadequate or impaired absorption and assimilation in patients; *^d^*inflammation associated with acute disease/injury or chronic disease; *In this study, patients diagnosed with Crohn’s disease were all thought to meet the etiologic criterion as GLIM consensus mentioned that most chronic organ diseases, such as Crohn’s disease, are associated with reduced food intake or impaired absorption and assimilation.

Patients were assessed for malnutrition using the GLIM criteria, which used a two-step model for the diagnosis. The first step was to identify at-risk status by the use of any validated screening tool. Second, patients who screen positive in the first step receive further screening to identify the malnutrition with GLIM (Figure 1).

In our study, we used NRS-2002 or MUST as the first step to identify patients who were at nutritional or malnutrition risk. NRS-2002 consists of the severity of disease (mild, moderate, or severe), impaired nutritional status which was scored according to the BMI, weight loss or reduced food intake, and the age with 70 years old as the dividing line. The final score of NRS-2002 ranges from 0 to 7. A score of 3 to 7 indicated that the patient had the nutritional risk (18), which was recorded as NRS-2002 (+). MUST was used for malnutrition risk screening, according to the ESPEN guidelines (11). It formulates a risk of malnutrition score based on current BMI (0 point with the BMI > 20 kg/m^2^; 1 with the BMI 18.5–20 kg/m^2^; 2 with the BMI < 18.5 kg/m^2^), involuntary weight loss in the past 3-6 months (0 point with the weight loss < 5%; 1 with the weight loss 5–10%; 2 with the weight loss > 10%), and suffering from acute disease or without nutritional intake for 5 days (2 points if either of them applies). MUST score ≥ 1 was considered of malnutrition risk, which is recorded as MUST (+).

Patients who were screened positive in the first step received further assessment. In the second step of GLIM, phenotype criteria include: (1) involuntary weight loss: > 5% within 6 months or > 10% beyond 6 months; (2) low BMI for Asian: < 18.5 kg/m^2^ if < 70 years or < 20.0 kg/m^2^ if > 70 years; and (3) reduced muscle mass: a low fat-free muscle index (FFMI) (< 15 kg/m^2^ for women, < 17 kg/m^2^ for men) or a low appendicular skeletal muscle mass index (ASMI) (< 5.7 kg/m^2^ for women, < 7.0 kg/m^2^ for men). Etiologic criteria included reduced food intake or assimilation, and disease burden or inflammation. As GLIM consensus mentioned that most chronic organ diseases, such as CD, are associated with reduced food intake or impaired absorption and assimilation, patients diagnosed with CD in this study were all thought to meet the etiologic criterion. To figure out malnutrition with GLIM, at least one phenotype criterion and one etiologic criterion should be presented (9). And then patients were divided into malnourished and well-nourished cohorts assessed by GLIM criteria using NRS-2002 and MUST as the first step, respectively.

Patients also received malnutrition assessment of PG-SAG. PG-SGA consists of two sections: the first includes the questionnaire about recent weight loss, food intake, symptoms that could interfere with food intake, and the physical activity level of the patients. For the second section, information is collected about the relationship between the disease and nutritional needs, metabolic requirement, and physical examination of the patient. Each item noted above has a separate score, and scores for each of the parameters were added up and given a total score. A total score greater than or equal to 4 was defined as malnutrition and recorded as PG-SGA (+).

Body composition was measured by InBody S10 analyzer (Biospace Co., Ltd., Seoul, Korea), which relied upon bioelectrical impedance analysis (BIA) technology, using an eight-point tetrapolar electrode system method which assesses the impedance to six specific frequencies (1, 5, 50, 250, 500, and 1,000 kHz) and the impedance at three specific frequencies (5, 50, and 250 kHz) of a small alternate electrical current applied on the body. The height and the body weight were measured at shoes-free and fasting status, and the BMI was calculated. Eventually, data including weight, body mass, BMI, fat-free mass (FFM), appendicular skeletal muscle mass (ASM), and fat mass (FM) could be obtained. The FFMI and ASMI were calculated by dividing a patient’s FFM or ASM values by the height squared (m^2^). ASMI = ASM/(height × height), FFMI = FFM/(height × height), body fat percent (BFP) = body fat mass/body weight × 100%, and body cell mass (BCM) = intracellular water + protein. The threshold for low muscle mass in men is ASMI < 7 kg/m^2^ or FFMI < 17 kg/m^2^, and in women is ASMI < 5.7 kg/m^2^ or FFMI < 15 kg/m^2^ (9).

### 2.4. Statistical analysis

Statistical analysis was performed with SPSS version 23.0 (Statistical Product and Service Solutions, CA, USA). Variables were subjected to test for normal distribution by the Kolmogorov–Smirnov test. The variables with normal distribution were expressed as means and standard deviation (SD). The variables with non-normal distribution were expressed as median and interquartile range (IQR). The variables with normal distribution were compared with t-test, while the variables with non-parametric distribution were compared with Mann–Whitney U test. The chi-square test was used for categorical variables. A value of *p* < 0.05 was considered as statistically significant.

## 3. Results

### 3.1. General characteristics

A total of 146 CD patients (121 males, 25 females) were recruited into the present study. All the patients were younger than 70 years old. The mean BMI was 17.72 ± 2.29, and 63.0% (92/146) of the patients had a BMI lower than 18.5 kg/m^2^. The mean albumin and hemoglobin were 37.06 ± 4.00 and 120.14 ± 18.00, respectively (Table 1).

**TABLE 1:** Basic information, body composition analysis and subtypes of the Crohn’s disease patients (*n* = 146).

Variables	Values
Basic information	
Gender, *n* (%)	
Male	121 (82.9)
Female	25 (17.1)
Age (years)	29.79 ± 7.00
Height (cm)	1.69 ± 0.07
Weight (kg)	49.85 ± 9.70
BMI (kg/m^2^)	17.72 ± 2.29
<18.5 kg/m^2^, *n* (%)	92 (63.0)
≥ 18.5, ≤ 20.0 kg/m^2^, *n* (%)	31 (21.2)
>20.0kg/m^2^, *n* (%)	23 (15.8)
Albumin (g/L)	37.06 ± 4.00
Hemoglobin (g/L)	120.14 ± 18.00
Body composition analysis	
ASMI (kg/m^2^)	6.59 ± 0.91
Normal ASMI, *n* (%)	68 (46.6)
Male (≥7.0kg/m^2^)	7.45 ± 0.31
Female (≥5.7 kg/m^2^)	5.96 ± 0.15
Low ASMI, *n* (%)	78 (53.4)
Male (<7.0kg/m^2^)	6.50 ± 0.45
Female (<5.7 kg/m^2^)	5.02 ± 0.54
FFMI (kg/m^2^)	15.35 ± 1.69
Normal FFMI, *n* (%)	33 (22.6)
Male (≥ 17.0kg/m^2^)	17.66 ± 0.57
Female (≥ 15.0 kg/m^2^)	15.47 ± 0.38
Low FFMI, *n* (%)	113 (77.4)
Male (<17.0kg/m^2^)	15.37 ± 1.42
Female (<15.0 kg/m^2^)	13.43 ± 1.12
BFP (%)	10.80 ± 9.00
Male	10.74 ± 5.13
Female	19.80 ± 6.59
BCM (kg)	28.53 ± 4.73
Subtypes of Crohn’s disease	
Age (years), *n* (%)	
A1 (≤ 16)	0 (0.0)
A2 (17–40)	135 (92.5)
A3 (>40)	11 (7.5)
Location, *n* (%)	
L1 (ileal)	63 (43.2)
L2 (colonic)	21 (14.4)
L3 (ileocolonic)	52 (35.6)
L4 (isolated upper disease)	10 (6.8)
Disease behavior, *n* (%)	
B1 (non-penetrating, non-stricturing)	13 (8.9)
B2 (stricturing)	97 (66.5)
B3 (penetrating)	30 (20.5)
P (perianal disease modifier)	6 (4.1)

BMI, body mass index; ASMI, appendicular skeletal muscle mass index; FFMI, fat-free mass index; BFP, body fat percent; BCM, body cell mass. The variables of BMI, ASMI, ASMI for male and female, FFMI, FFMI for male and female, BFP for male and female, and BCM were expressed as means ± standard deviation (SD). The variables of Age, Height, Weight, Albumin, Hemoglobin, and BFP were expressed as median ± Inter Quartile Range (IQR). Categorical variables were presented as absolute values and percentages (*n*, %).

More than half (53.4%, 78/146) of the patients had a low ASMI (male < 7.0 kg/m^2^, female < 5.7 kg/m^2^), with the mean ASMI of 6.50 ± 0.45 kg/m^2^ for male and 5.02 ± 0.54 kg/m^2^ for female. More than two-thirds (77.4%, 113/146) of the patients had a low FFMI (male < 17.0 kg/m^2^, female < 15.0 kg/m^2^), with the mean FFMI of 15.37 ± 1.42 kg/m^2^ for male and 13.43 ± 1.12 kg/m^2^ for female. The mean BFP (%) was 10.80 ± 9.00, with the mean BFP(%) of 10.74 ± 5.13 for male and 19.80 ± 6.59 for female. And the mean BCM (kg) was 28.53 ± 4.73 (Table 1).

In the present study, more than four-fifths (92.5%, 135/146) of the CD patients’ age were between 17 and 40 years old. Almost half (43.2%, 63/146) of the patients’ lesion site was in ileal, and almost two-thirds (66.4%, 97/146) of the patients’ disease behavior was stricturing (Table 1).

There was 62.3% (91/146) of the patients had nutritional risk when screening by NRS-2002 (≥ 3 scores) and 89.7% (131/146) had malnutrition risk after screening by MUST (≥ 1 scores). There was 67.8% (99/146) of the patients had malnutrition after assessed with PG-SGA (56% moderate/suspected malnutrition according to PG-SGA of 4–8 scores and 17% severe malnutrition according to PG-SGA of ≥ 9 scores) (Table 2).

**TABLE 2:** The scores of the different nutrition assessment tools (*n* = 146).

Nutrition assessment scores	*n* (%)
NRS-2002 score	146
No nutritional risk (score < 3)	55 (37.7)
Nutritional risk (score ≥ 3)	91 (62.3)
MUST score	146
No malnutrition risk (score < 1)	15 (10.3)
Malnutrition risk (score ≥ 1)	131 (89.7)
PG-SGA score	146
No malnutrition (score < 4)	47 (32.2)
Malnutrition (score ≥ 4)	99 (67.8)
Moderate/suspected malnutrition (score of 4-8)	75 (75.8)
Severe malnutrition (score of ≥ 9)	24 (24.2)

NRS-2002, nutrition risk screening 2002; MUST, malnutrition universal screening tool; PG-SGA, patient-generated subjective global assessment.

### 3.2. Comparisons of characteristics between the malnourished and well-nourished patients assessed by GLIM criteria using NRS-2002 and MUST as the first step, respectively

The prevalence of malnutrition was 59.6% (87/146) and 82.2% (120/146) in the cohort of GLIM defined malnutrition using NRS-2002 and MUST as the first step, respectively. In the two cohorts, there were all no significant differences in age between the malnourished and well-nourished patients (*p* = 0.64 and 0.08, respectively), and the BMI, BCM, BFP, ASMI, and FFMI were all significantly lower in the malnourished patients than in normal nourished patients (*p* were all < 0.05) (Table 3).

**TABLE 3:** Comparisons of characteristics between the malnourished and well-nourished patients assessed by GLIM criteria using NRS-2002 and MUST as the first step, respectively (*n* = 146).

Characteristics	GLIM defined malnutrition using NRS-2002 as the first step			GLIM defined malnutrition using MUST as the first step		
	**Malnourished (*n* = 87)**	**Well-nourished** **(*n* = 59)**	***P***	**Malnourished (*n* = 120)**	**Well-nourished (*n* = 26)**	***p***
Age (years)	29.76 ± 0.88	29.83 ± 7.20	0.64	29.33 ± 8.12	31.92 ± 8.00	0.08
BMI (kg/m^2^)	16.91 ± 1.96	19.15 ± 1.95	<0.001	17.15 ± 1.81	20.88 ± 1.31	<0.001
BCM	27.96 ± 4.03	30.26 ± 4.71	<0.001	28.02 ± 4.00	32.91 ± 4.26	<0.001
BFP (%)	10.66 ± 5.85	14.70 ± 6.41	0.000	11.25 ± 6.28	17.08 ± 4.43	0.000
ASMI (kg/m^2^)	6.51 ± 0.82	6.95 ± 0.83	<0.001	6.55 ± 0.83	7.34 ± 0.64	<0.001
FFMI (kg/m^2^)	15.01 ± 1.57	16.26 ± 1.36	<0.001	15.17 ± 1.45	17.28 ± 0.93	<0.001

GLIM, Global Leader Initiative on Malnutrition; BMI, body mass index; BCM, body cell mass; BFP, body fat percent; ASMI, appendicular skeletal; FFMI, fat-free mass index. The BMI, BCM, ASMI, and FFMI between the malnourished and well-nourished groups were expressed as means ± standard deviation (SD). The Age and BFP(%) between the malnourished and well-nourished groups were expressed as median ± Inter Quartile Range (IQR). *p*-value < 0.05 refers to the significant difference between malnourished group and well-nourished group.

### 3.3. Comparisons of basic characteristics between the patients with NRS-2002 (−) & MUST (+) and the patients with NRS-2002 (−) & MUST (−)

There were 55 patients who had no nutritional risk when screening by NRS-2002 (< 3 scores), 41 (74.5%, 41/55) of whom had a MUST score greater than or equal to 1 (MUST +), and 14 (25.5%, 14/55) of whom had a MUST score lower than 1 (MUST -). Between the patients with NRS-2002 (−) & MUST (+) and the patients with NRS-2002 (−) & MUST (−), there were no significant differences in age regardless of the age subgroup (*p* = 0.20 and 1.0, respectively), and the BMI, SMI, and FFMI were all significantly lower in the former group (*p* were all < 0.05) (Table 4).

**TABLE 4:** Comparisons of basic characteristics between the patients with NRS-2002 (−) & MUST (+) and the patients with NRS-2002 (−) & MUST (−) (*n* = 55).

Characteristics	NRS-2002 (−) & MUST (+) *n* = 41	NRS-2002 (−) & MUST (−) *n* = 14	*p*
Age (years old)	28.37 ± 5.63	32.00 ± 9.69	0.20
≥ 70	0	0	1.0
< 70	41	14	
BMI(kg/m^2^)	18.12 ± 1.22	21.45 ± 1.13	<0.001
< 18.5	25	0	<0.001
18.5–20.0	15	0	
> 20.0	1	14	
ASMI (kg/m^2^)	6.71 ± 0.84	7.48 ± 0.52	<0.001
FFMI (kg/m^2^)	15.69 ± 1.14	17.55 ± 0.89	<0.001

BMI, body mass index; ASMI, appendicular skeletal muscle mass index; FFMI, fat-free mass index. The variables of Age, BMI, ASMI, and FFMI were expressed as means and SD. Age categories and BMI categories were expressed as n. *p*-value < 0.05 refers to the significant difference between two group.

In order to further investigate which nutritional risk screening test was most convenient to use in order to diagnose a greater number of patients with malnutrition and have an earlier approach in this population that already has a higher risk of having malnutrition. The comparisons of characteristics between the malnourished patients assessed by GLIM criteria using NRS-2002 and MUST as the first step, respectively, were analyzed, and none of the characteristics were statistically significant (*p* were all > 0.05) (Supplementary Table 1).

### 3.4. Characteristics of patients with NRS-2002 (−) and MUST (+)

There were 41 patients with NRS-2002 (−) and MUST (+). Among them, 39.0% (16/41) patients had a MUST score of 1 point, with 93.8% (15/16) scoring due to BMI of 18.5∼20.0 kg/m^2^, only 1 due to the unplanned weight loss of 5-10% in the past 3–6 months. There were 61.0% (25/41) patients who had a MUST score greater than or equal to 2 points due to BMI < 18.5 kg/m^2^. Slightly more than one-third (39.0%, 16/41) and more than four-fifths (80.5%, 33/41) of the patients were diagnosed as malnutrition assessed by PG-SGA and GLIM, respectively. Among the 41 patients, slightly less than one-third (31.7%, 13/41) had a low ASMI, 61.5% (8/13) of whom were male and 38.5% (5/13) of whom were female. Almost four-fifths (78.0%, 32/41) had a low FFMI, 78.1% (25/32) of whom were male and 21.9% (7/32) were female (Table 5).

**TABLE 5:** Characteristics of patients with NRS-2002 (−) and MUST (+) (*n* = 41).

Characteristics	*n* (%)
MUST score	41
Malnutrition risk (score ≥ 1)	41
Score of 1	16 (39.0)
BMI of 18.5∼20.0 kg/m2	15 (93.8)
Weight loss of 5-10% in the past 3–6 months	1 (6.2)
Score of ≥ 2	25 (61.0)
BMI < 18.5 kg/m^2^	25 (100.0)
PG-SGA score	41
Well-nourished (<4)	25 (61.0)
Malnourished (≥4)	16 (39.0)
Moderate/suspected malnutrition (score of 4–8)	16 (100.0)
Severe malnutrition (score of ≥ 9)	0 (0.0)
GLIM	41
Well-nourished	8 (19.5)
Malnourished	33 (80.5)
ASMI	41
Normal ASMI	28 (68.3)
Low ASMI	13 (31.7)
Male (<7.0 kg/m^2^)	8 (61.5)
Female (<5.7 kg/m^2^)	5 (38.5)
FFMI	41
Normal FFMI	9 (22.0)
Low FFMI	32 (78.0)
Male (<17.0 kg/m^2^)	25 (78.1)
Female (<15.0 kg/m^2^)	7 (21.9)

MUST, malnutrition universal screening tool; MUST score equal or greater than 1 means MUST (+); PG-SGA, patient-generated subjective global assessment; GLIM, Global Leader Initiative on Malnutrition; ASMI, appendicular skeletal muscle mass index; FFMI, fat-free mass index.

## 4. Discussion

The nutritional status of CD patients is not optimistic. There exists a high prevalence of nutritional risk or even malnutrition among CD patients. Y Wu et al. assessed the nutritional risk in CD patients using NRS-2002 and found the prevalence was 65.2% (464/712) (7). In our study, we found the prevalence of nutritional and malnutrition risk among CD inpatients is as high as 62.3% (91/146) according to NRS-2002 and 89.7% (131/146) according to MUST. Studies also showed that the rate of malnutrition is between 20% and 85% in CD patients, depending on different malnutrition criteria (19). Our study showed that 67.8% (99/146) of the inpatients had malnutrition according to PG-SGA and that 59.6% (87/146) or 82.2% (120/146) of the inpatients had malnutrition according to GLIM after using NRS-2002 or MUST as the first screening step, respectively, which means the prevalence of malnutrition in CD patients is more than 50% regardless of which assessment tool was used.

Malnourished patients with CD are more likely to be hospitalized following emergency department attendance and more likely to be admitted to the hospital due to infection (20, 21). In hospitalized patients, malnutrition is an independent risk factor for venous thromboembolism, non-elective surgery, longer admission, and increased mortality (22–24). Moreover, sarcopenia is associated with malnutrition (25) and often occurred in CD patients. Due to a significant deficiency in fat-free mass and muscle strength observed in CD patients compared with the healthy controls, it was advised that more parameters should be included in nutrition assessment besides weight and BMI (26). We selected ASMI and FFMI to assess the nutritional status of CD patients and found that 53.4% (78/146) or 77.4% (113/146) of the patients had low ASMI or FFMI, which suggested that more than half of the CD inpatients had a reduced muscle mass. Patients with malnutrition assessed by GLIM had significantly lower ASMI and FFMI than patients with normal nutritional status, which indicated that CD patients may be accompanied by the loss of muscle mass once they were diagnosed with malnutrition. Therefore, in addition to the commonly used indicators, it is necessary to add quantifiable indicators to assess the nutritional status of CD patients, such as FFMI and ASMI.

Laboratory tests of serum proteins, such as albumin and hemoglobin, are frequently used to assess protein malnutrition in clinical practice. However, our results showed that only 14.4% (21/146) of the CD inpatients had hypoalbuminemia and no patients had hypo-hemoglobinemia, suggesting that albumin and hemoglobin cannot be used as the only indicator for nutritional assessment.

As for GLIM, the diagnosis of malnutrition includes screening and assessment. NRS-2002 is the most widely used as the first step of GLIM. Are there other tools that can be used as the first step of GLIM? Xu et al. (27) first used MNA-SF as the nutritional screening tool for elderly patients as the first step of GLIM. In adults, risk of malnutrition of IBD patients can be assessed with MUST, which has also been validated (28–30).

In our study, nutritional risk screening was performed in both MUST and NRS-2002, and it showed that 28.1% (41/146) was still identified as malnutrition risk through MUST as NRS-2002 evaluated no nutritional risk. Among the 41 patients, we found that 80.5% (33/41) were defined as malnutrition by GLIM; meanwhile, 31.7% (13/41) and 78.0% (32/41) were with low ASMI and FFMI, respectively. It suggested that NRS-2002 may miss some patients with nutritional risk or sarcopenia, for which MUST may be used as a supplement to NRS-2002.

NRS-2002 and MUST have similarities and differences. The similarities are that both of them contain the BMI, weight loss, and disease status. The differences, which may lead to the result of NRS-2002(−) but MUST (+), are as follows. First, the cut-off value of BMI is different. MUST defines BMI of 18.5–20.0 kg/m^2^ as medium risk, without paying attention to the general condition of the patients. However, NRS-2002 defines BMI relatively strictly, requiring a BMI < 18.5 kg/m^2^ combined with impaired general condition. Our study showed that 36.5% (15/41) of the patients were diagnosed as malnutrition risk by MUST due to BMI 18.5–20.0 kg/m^2^ and 61.0% (25/41) due to BMI < 18.5 kg/m^2^ without involved poor general conditions. Second, the rate of unplanned weight loss and the time range are different. The rate of weight loss in NRS-2002 is fixed at 5%, and the time range is 3 months, 2 months, and 1 month, respectively. The shorter the time, the higher the score. However, the time range in MUST was fixed in the past 3–6 months, with the weight loss rates of <5%, 5-10%, and >10%, respectively, which makes some patients with a weight loss rate >5% in the past 6 months but with no weight loss in the past 3 months are rated as medium malnutrition risk by MUST but are rated as no nutritional risk by NRS-2002. There was one patient in our study fell into this category. Third, NRS-2002 covers the food intake, which may affect the nutritional status of the patients, and the age criteria, i.e., ≥ 70 years old can get 1 extra point, which MUST lacks. Since all the CD patients enrolled in our study were younger than 70 years old, it makes the advantage of NRS-2002 unable to reflect.

In conclusion, NRS-2002 and MUST have their own advantages and disadvantages. Combining the two may reduce the missing rate of the patients at potential malnutrition risk. Nutritional risk screening tools are recommended to be used flexibly for CD patients, especially as the patient has one of the characteristics as follows: (1) NRS-2002 shows no nutritional risk, but BMI is between 18.5 kg/m^2^ and 20.0 kg/m^2^. (2) BMI < 18.5 kg/m^2^ without poor general conditions. (3) Patients with low ASMI or FFMI.

## 5. Limitations of the study

The ASMI used in the assessment of muscle mass reduction refers to the Asian Working Group for Sarcopenia for Asians (AWGS) standard, while FFMI is applicable to European populations. The absence of Chinese cut-off reduces the applicability of the study and requires more evidence-based medical evidence. However, we revealed that MUST can be used as the first step in CD patients with an NRS-2002 score less than 3, which prevented the entrance of the second step of GLIM.

## Data availability statement

The raw data supporting the conclusions of this article will be made available by the authors, without undue reservation.

## Ethics statement

The studies involving human participants were reviewed and approved by the Institutional Review Boards of the Third Xiangya Hospital, Central South University (2019-S540). The Ethics Committee waived the requirement of written informed consent for participation.

## Author contributions

HL and ML designed the study and were the guarantors of this work. MW, CT, QG, JW, and GF conducted the study and analyzed and interpreted the data. MW wrote the manuscript. MW, GF, and WW performed the data collection. All authors approved the final version of this manuscript and agreed to be accountable for the content of the work.
