# Supplementary material for: GLIM criteria using NRS-2002 and MUST as the first step adequately diagnose the malnutrition in Crohn’s disease inpatients: A retrospective study
Source: Front Nutr. 2023 Jan 11;9:1059191. doi: 10.3389/fnut.2022.1059191 (PMC9874672; doi:10.3389/fnut.2022.1059191)
Supplement: Supplementary file 1 [file Table_1.DOCX]

Supplementary Table 1. Comparisons of characteristics between the malnourished patients assessed by GLIM criteria using NRS-2002 and MUST as the first step, respectively(n=146).

| **Characteristics** | **GLIM defined malnutrition using NRS-2002 as the first step**  **(n=87)** | **GLIM defined malnutrition using MUST as the first step**  **(n=120)** | ***p*** |
| --- | --- | --- | --- |
| Age (years) | 29.76(0.88) | 29.33(8.12) | 0.81 |
| BMI (kg/m^2^) | 16.91(1.96) | 17.15(1.81) | 0.37 |
| BCM | 27.96(4.03) | 28.02(4.00) | 0.73 |
| BFP (%) | 10.66(5.85) | 11.25(6.28) | 0.55 |
| ASMI (kg/m^2^) | 6.51(0.82) | 6.55(0.83) | 0.52 |
| FFMI (kg/m^2^) | 15.01(1.57) | 15.17(1.45) | 0.60 |

GLIM, Global Leader Initiative on Malnutrition; BMI, body mass index; BCM, body cell mass; BFP, body fat percent; ASMI, appendicular skeletal; FFMI, fat-free mass index.The Age, BMI, BCM, BFP, ASMI and FFMI between the two malnourished groups were expressed as means and standard deviation (SD). *p* value <0.05 refers to the significant difference between malnourished group and well-nourished group.
